# #COVIDisAirborne: AI-Enabled Multiscale Computational Microscopy of Delta SARS-CoV-2 in a Respiratory Aerosol

**DOI:** 10.1101/2021.11.12.468428

**Published:** 2021-11-15

**Authors:** Abigail Dommer, Lorenzo Casalino, Fiona Kearns, Mia Rosenfeld, Nicholas Wauer, Surl-Hee Ahn, John Russo, Sofia Oliveira, Clare Morris, Anthony Bogetti, Anda Trifan, Alexander Brace, Terra Sztain, Austin Clyde, Heng Ma, Chakra Chennubhotla, Hyungro Lee, Matteo Turilli, Syma Khalid, Teresa Tamayo-Mendoza, Matthew Welborn, Anders Christensen, Daniel G. A. Smith, Zhuoran Qiao, Sai Krishna Sirumalla, Michael O’Connor, Frederick Manby, Anima Anandkumar, David Hardy, James Phillips, Abraham Stern, Josh Romero, David Clark, Mitchell Dorrell, Tom Maiden, Lei Huang, John McCalpin, Christopher Woods, Alan Gray, Matt Williams, Bryan Barker, Harinda Rajapaksha, Richard Pitts, Tom Gibbs, John Stone, Daniel Zuckerman, Adrian Mulholland, Thomas Miller, Shantenu Jha, Arvind Ramanathan, Lillian Chong, Rommie Amaro

**Affiliations:** 1 UC San Diego,; 2 Oregon Health & Science University,; 3 University of Bristol,; 4 University of Pittsburgh,; 5 Argonne National Laboratory,; 6 University of Illinois at Urbana-Champaign,; 7 University of Chicago,; 8 Freie Universität Berlin,; 9 Brookhaven National Lab & Rutgers University; 10 University of Oxford,; 11 Entos, Inc.,; 12 California Institute of Technology,; 13 NVIDIA Corporation,; 14 Pittsburgh Supercomputing Center,; 15 Texas Advanced Computing Center,; 16 Oracle for Research

**Keywords:** molecular dynamics, deep learning, multiscale simulation, weighted ensemble, computational virology, SARS-CoV-2, aerosols, COVID-19, HPC, AI, GPU, Delta

## Abstract

We seek to completely revise current models of airborne transmission of respiratory viruses by providing never-before-seen atomic-level views of the SARS-CoV-2 virus within a respiratory aerosol. Our work dramatically extends the capabilities of multiscale computational microscopy to address the significant gaps that exist in current experimental methods, which are limited in their ability to interrogate aerosols at the atomic/molecular level and thus obscure our understanding of airborne transmission. We demonstrate how our integrated data-driven platform provides a new way of exploring the composition, structure, and dynamics of aerosols and aerosolized viruses, while driving simulation method development along several important axes. We present a series of initial scientific discoveries for the SARS-CoV-2 Delta variant, noting that the full scientific impact of this work has yet to be realized.

## 1 JUSTIFICATION

We develop a novel HPC-enabled multiscale research framework to study aerosolized viruses and the full complexity of species that comprise them. We present technological and methodological advances that bridge time and length scales from electronic structure through whole aerosol particle morphology and dynamics.

## 2 PERFORMANCE ATTRIBUTES

**Table:** 

Performance Attribute	Our Submission
Category of achievement	Scalability, Time-to-solution
Type of method used	Explicit, Deep Learning
Results reported on the basis of	Whole application including I/O
Precision reported	Mixed Precision
System scale	Measured on full system
Measurement mechanism	Hardware performance counters, Application timers, Performance Modeling

## 3 OVERVIEW OF THE PROBLEM

**Figure 1:**
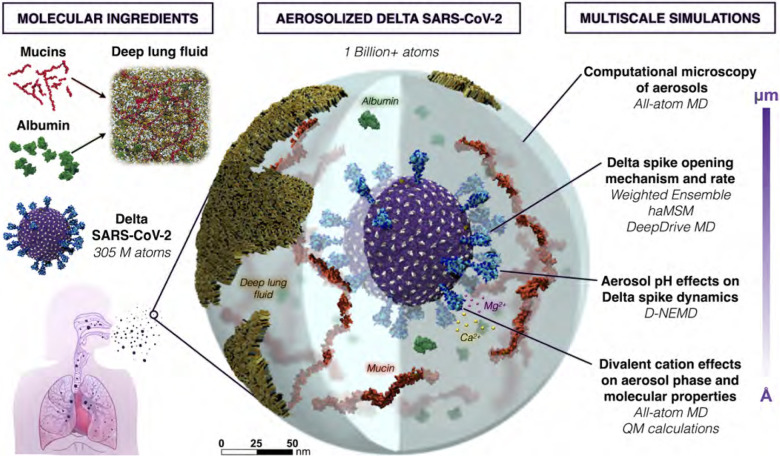
Overall schematic depicting the construction and multiscale simulations of Delta SARS-CoV-2 in a respiratory aerosol. (N.B.: The size of divalent cations has been increased for visibility.)

Respiratory pathogens, such as SARS-CoV-2 and influenza, are the cause of significant morbidity and mortality worldwide. These respiratory pathogens are spread by virus-laden aerosols and droplets that are produced in an infected person, exhaled, and transported through the environment (Wang et al., 2021) (Fig 1). Medical dogma has long focused on droplets as the main transmission route for respiratory viruses, where either a person has contact with an infected surface (fomites) or direct droplet transmission by close contact with an infected individual. However, as we continue to observe with SARS-CoV-2, airborne transmission also plays a significant role in spreading disease. We know this from various super spreader events, e.g., during a choir rehearsal (Miller et al., 2021). Intervention and mitigation decisions, such as the relative importance of surface cleaning or whether and when to wear a mask, have unfortunately hinged on a weak understanding of aerosol transmission, to the detriment of public health.

A central challenge to understanding airborne transmission has been the inability of experimental science to reliably probe the structure and dynamics of viruses once they are inside respiratory aerosol particles. Single particle experimental methods have poor resolution for smaller particles (<1 micron) and are prone to sample destruction during collection. Airborne viruses are present in low concentrations in the air and are similarly prone to viral inactivation during sampling. In addition, studies of the initial infection event, for example in the deep lung, are limited in their ability to provide a detailed understanding of the myriad of molecular interactions and dynamics taking place *in situ*. Altogether, these knowledge gaps hamper our collective ability to understand mechanisms of infection and develop novel effective antivirals, as well as prevent us from developing concrete, science-driven mitigation measures (e.g., masking and ventilation protocols).

Here, we aim to reconceptualize current models of airborne transmission of respiratory viruses by providing never-before-seen views of viruses within aerosols. Our approach relies on the use of all-atom molecular dynamics (MD) simulations as a multiscale ‘computational microscope.’ MD simulations can synthesize multiple types of biological data (e.g., multiresolution structural datasets, glycomics, lipidomics, etc.) into cohesive, biologically ‘accurate’ structural models. Once created, we then approximate the model down to its many atoms, creating trajectories of its time dependent dynamics under cell-like (or in this case, aerosol-like) conditions. Critically, MD simulations are more than just ‘pretty movies.’ MD equations are solved in a theoretically rigorous manner, allowing us to compute experimentally testable macroscopic observables from time-averaged microscopic properties. What this means is that we can directly connect MD simulations with experiments, each validating and providing testable hypotheses to the other, which is the real power of the approach. An ongoing challenge to the successful application of such methods, however, is the need for technological and methodological advances that make it possible to access length scales relevant to the study of large, biologically complex systems (spanning nanometers to õne micron in size) and, correspondingly, longer timescales (microseconds to seconds).

Such challenges and opportunities manifest in the study of aerosolized viruses. Aerosols are generally defined as being less than 5 microns in diameter, able to float in the air for hours, travel significant distances (e.g., can fill a room, like cigarette smoke), and be inhaled. Fine aerosols < 1 micron in size can stay in the air for over 12 hours and are enriched with viral particles (Coleman et al., 2021, Fennelly, 2020). Our work focuses on these finer aerosols that travel deeper into the respiratory tract. Several studies provide the molecular recipes necessary to reconstitute respiratory aerosols according to their actual biologically-relevant composition (Vejerano and Marr, 2018, Walker et al., 2021). These aerosols can contain lipids, cholesterol, albumin (protein), various mono- and di-valent salts, mucins, other surfactants, and water (Fig 1). Simulations of aerosolized viruses embody a novel framework for the study of aerosols: they will allow us and others to tune different species, relative humidity, ion concentrations, etc. to match experiments that can directly and indirectly connect to and inform our simulations, as well as test hypotheses. Some of the species under study here, e.g., mucins, have not yet been structurally characterized or explored with simulations and thus the models we generate are expected to have impact beyond their roles in aerosols.

**Figure 2:**
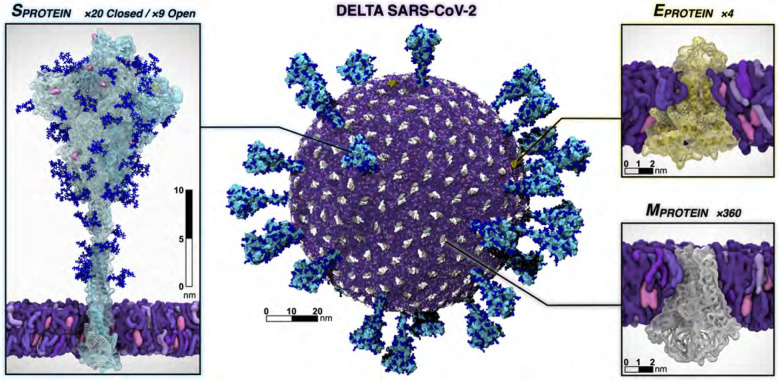
Individual protein components of the SARS-CoV-2 Delta virion. The spike is shown with the surface in cyan and with Delta’s mutated residues and deletion sites highlighted in pink and yellow, respectively. Glycans attached to the spike are shown in blue. The E protein is shown in yellow and the M protein is shown in silver and white. Visualized with VMD.

In addition to varying aerosol composition and size, the viruses themselves can be modified to reflect new variants of concern, where such mutations may affect interactions with particular species in the aerosol that might affect its structural dynamics and/or viability. The virion developed here is the Delta variant (B.1.617.2 lineage) of SARS-CoV-2 (Fig 2), which presents a careful integration of multiple biological datasets: (1) a complete viral envelope with realistic membrane composition, (2) fully glycosylated full-length spike proteins integrating 3D structural coordinates from multiple cryoelectron microscopy (cryoEM) studies (Bangaru et al., 2020, McCallum et al., 2021, Walls et al., 2020, Wrapp et al., 2020) (3) all biologically known features (post-translational modifications, palmitoylation, etc.), (4) any other known membrane proteins (e.g., the envelope (E) and membrane (M) proteins), and (5) virion size and patterning taken directly from cryoelectron tomography (cryoET). Each of the individual components of the virus are built up before being integrated into the composite virion, and thus represent useful molecular-scale scientific contributions in their own right (Casalino et al., 2020, Sztain et al., 2021).

Altogether in this work, we dramatically extend the capabilities of data-driven, multiscale computational microscopy to provide a new way of exploring the composition, structure, and dynamics of respiratory aerosols. While a seemingly limitless number of putative hypotheses could result from these investigations, the first set of questions we expect to answer are: *How does the virus exist within a droplet of the same order of magnitude in size, without being affected by the air-water interface, which is known to destroy molecular structure * (D’Imprima et al., 2019) *? How does the biochemical composition of the droplet, including pH, affect the structural dynamics of the virus? Are there species within the aerosols that “buffer” the viral structure from damage, and are there particular conditions under which the impact of those species changes?* Our simulations can also provide specific parameters that can be included in physical models of aerosols, which still assume a simple water or water-salt composition even though it is well known that such models, e.g., using kappa-Kohler theory, break down significantly as the molecular species diversify (Petters and Kreidenweis, 2007).

## 4 CURRENT STATE OF THE ART

Current experimental methods are unable to directly interrogate the atomic-level structure and dynamics of viruses and other molecules within aerosols. Here we showcase computational microscopy as a powerful tool capable to overcome these significant experimental limitations. We present the major elements of our multiscale computational microscope and how they come together in an integrated manner to enable the study of aerosols across multiple scales of resolution. We demonstrate the impact such methods can bring to bear on scientific challenges that until now have been intractable, and present a series of new scientific discoveries for SARS-CoV-2.

### 4.1 Parallel molecular dynamics

All-atom molecular dynamics simulation has emerged as an increasingly powerful tool for understanding the molecular mechanisms underlying biophysical behaviors in complex systems. Leading simulation engines, NAMD (Phillips et al., 2020), AMBER (Case et al.), and GROMACS (Páll et al., 2020), are broadly useful, with each providing unique strengths in terms of specific methods or capabilities as required to address a particular biological question, and in terms of their support for particular HPC hardware platforms. Within the multiscale computational microscopy platform developed here, we show how each of these different codes contributes different elements to the overall framework, oftentimes utilizing different computing modalities/architectures, while simultaneously extending on state-of-the-art for each. Structure building, simulation preparation, visualization, and post-hoc trajectory analysis are performed using VMD on both local workstations and remote HPC resources, enabling modeling of the molecular systems studied herein (Humphrey et al., 1996, Sener et al., 2021, Stone et al., 2013a,b, 2016b). We show how further development of each of these codes, considered together within the larger-scale collective framework, enables the study of SARS-CoV-2 in a wholly novel manner, with extension to numerous other complex systems and diseases.

### 4.2 AI-enhanced WE simulations

Because the virulence of the Delta variant of SARS-CoV-2 may be partly attributable to spike protein (S) opening, it is of pressing interest to characterize the mechanism and kinetics of the process. Although S-opening in principle can be studied via conventional MD simulations, in practice the system complexity and timescales make this wholly intractable. Splitting strategies that periodically replicate promising MD trajectories, among them the weighted ensemble (WE) method (Huber and Kim, 1996, Zuckerman and Chong, 2017), have enabled simulations of the spike opening of WT SARS-CoV-2 (Sztain et al., 2021, Zimmerman et al., 2021). WE simulations can be orders of magnitude more efficient than conventional MD in generating pathways and rate constants for rare events (e.g., protein folding (Adhikari et al., 2019) and binding (Saglam and Chong, 2019)). The WESTPA software for running WE (Zwier et al., 2015) is well-suited for high-performance computing with nearly perfect CPU/GPU scaling. The software is interoperable with any dynamics engine, including the GPU-accelerated AMBER dynamics engine (Salomon-Ferrer et al., 2013) that is used here. As shown below, major upgrades to WESTPA (v. 2.0) have enabled a dramatic demonstration of spike opening in the Delta variant (Figs. 5, 6) and exponentially improved analysis of spike-opening kinetics.

The integration of AI techniques with WE can further enhance the efficiency of sampling rare events (Brace et al., 2021b, Casalino et al., 2021, Noé, 2020). One frontier area couples unsupervised linear and non-linear dimensionality reduction methods to identify collective variables/progress coordinates in high-dimensional molecular systems (Bhowmik et al., 2018, Clyde et al., 2021). Such methods may be well suited for analyzing the aerosolized virus. Integrating these approaches with WE simulations is advantageous in sampling the closed → open transitions in the Delta S landscape (Fig. 5) as these unsupervised AI approaches automatically stratify progress coordinates (Fig. 5D).

### 4.3 Dynamical Non-Equilibrium MD

Aerosols rapidly acidify during flight via reactive uptake of atmospheric gases, which is likely to impact the opening/closing of the S protein (Vejerano and Marr, 2018, Warwicker, 2021). Here, we describe the extension of dynamical non-equilibrium MD (D-NEMD) (Ciccotti and Ferrario, 2016) to investigate pH effects on the Delta S. D-NEMD simulations (Ciccotti and Ferrario, 2016) are emerging as a useful technique for identifying allosteric effects and communication pathways in proteins (Galdadas et al., 2021, Oliveira et al., 2019), including recently identifying effects of linoleic acid in the WT spike (Oliveira et al., 2021b). This approach complements equilibrium MD simulations, which provide a distribution of configurations as starting points for an ensemble of short non-equilibrium trajectories under the effect of the external perturbation. The response of the protein to the perturbation introduced can then be determined using the Kubo-Onsager relation (Ciccotti and Ferrario, 2016, Oliveira et al., 2021a) by directly tracking the change in atomic positions between the equilibrium and non-equilibrium simulations at equivalent points in time (Oliveira et al., 2021a).

### 4.4 OrbNet

Ca^2+^ ions are known to play a key role in mucin aggregation in epithelial tissues (Hughes et al., 2019). Our RAV simulations would be an ideal case-study to probe such complex interactions between Ca^2+^, mucins, and the SARS-CoV-2 virion in aerosols. However, Ca^2+^ binding energies can be difficult to capture accurately due to electronic dispersion and polarization, terms which are not typically modeled in classical mechanical force fields. Quantum mechanical (QM) methods are uniquely suited to capture these subtle interactions. Thus, we set out to estimate the correlation in Ca^2+^ binding energies between CHARMM36m and quantum mechanical estimates enabled via AI with OrbNet. Calculation of energies with sufficient accuracy in biological systems can, in many cases, be adequately described with density functional theory (DFT). However, its high cost limits the applicability of DFT in comparison to fixed charge force-fields. To capture quantum quality energetics at a fraction of the computational expense, we employ a novel approach (OrbNet) based on the featurization of molecules in terms of symmetry-adapted atomic orbitals and the use of graph neural network methods for deep-learning quantum-mechanical properties (Qiao et al., 2020). Our method outperforms existing methods in terms of its training efficiency and transferable accuracy across diverse molecular systems, opening a new pathway for replacing DFT in large-scale scientific applications such as those explored here. (Christensen et al., 2021).

## 5 INNOVATIONS REALIZED

### 5.1 Construction and simulation of SARS-CoV-2 in a respiratory aerosol

Our approach to simulating the entire aerosol follows a composite framework wherein each of the individual molecular pieces is refined and simulated on its own before it is incorporated into the composite model. Simulations of each of the components are useful in their own right, and often serve as the basis for biochemical and biophysical validation and experiments (Casalino et al., 2020).

Throughout, we refer to the original circulating SARS-CoV-2 strain as “WT”, whereas all SARS-CoV-2 proteins constructed in this work represent the Delta variant (Fig. 2). All simulated membranes reflect mammalian ER-Golgi intermediate compartment (ERGIC) mimetic lipid compositions. VMD (Humphrey et al., 1996, Stone et al., 2016a), psfgen (Phillips et al., 2005), and CHARMM-GUI (Park et al., 2019) were used for construction and parameterization. Topologies and parameters for simulations were taken from CHARMM36m all-atom additive force fields (Beglov and Roux, 1994, Guvench et al., 2009, Han et al., 2018, Huang and Mackerell, 2013, Huang et al., 2017, Klauda et al., 2010, Venable et al., 2013). NAMD was used to perform MD simulations (Phillips et al., 2020), adopting similar settings and protocols as in (Casalino et al., 2020). All systems underwent solvation, charge neutralization, minimization, heating and equilibration prior to production runs. Refer to Table 1 for Abbreviations, PBC dimensions, total number of atoms, and total equilibration times for each system of interest.

#### 5.1.1 Simulating the SARS-CoV-2 structural proteins.

Fully glycosylated Delta spike (S) structures in open and closed conformations were built based on WT constructs from Casalino et al. (Casalino et al., 2020) with the following mutations: T19R, T95I, G142D, E156G, Δ157–158, L452R, T478K, D614G, P681R, and D950N (Kannan et al., 2021, McCallum et al., 2021). Higher resolved regions were grafted from PDB 7JJI (Bangaru et al., 2020). Additionally, coordinates of residues 128–167 – accounting for a drastic conformational change seen in the Delta variant S – graciously made available to us by the Veesler Lab, were similarly grafted onto our constructs (McCallum et al., 2021). Finally, the S proteins were glycosylated following work by Casalino et al. (Casalino et al., 2020). By incorporating the Veesler Lab’s bleeding-edge structure (McCallum et al., 2021) and highly resolved regions from 7JJI (Bangaru et al., 2020), our models represent the most complete and accurate structures of the Delta S to date. The S proteins were inserted into membrane patches and equilibrated for 3 × 110 ns. For nonequilibrium and weighted ensemble simulations, a closed S head (SH, residues 13–1140) was constructed by removing the stalk from the full-length closed S structure, then resolvated, neutralized, minimized, and subsequently passed to WE and D-NEMD teams. The M protein was built from a structure graciously provided by the Feig Lab (paper in prep). The model was inserted into a membrane patch and equilibrated for 700 ns. RMSD-based clustering was used to select a stable starting M protein conformation. From the equilibrated and clustered M structure, VMD’s Mutator plugin (Humphrey et al., 1996) was used to incorporate the I82T mutation onto each M monomer to arrive at the Delta variant M. To construct the most complete E protein model to-date, the structure was patched together by resolving incomplete PDBs 5X29 (Surya et al., 2018), 7K3G (Mandala et al., 2020) and 7M4R (Chai et al., 2021). To do so, the transmembrane domain (residues 8–38) from 7K3G were aligned to the the N-terminal domain (residues 1–7) and residues 39 to 68 of 5X29 and residues 69 to 75 of 7M4R by their C_*α*_ atoms. E was then inserted into a membrane patch and equilibrated for 40 ns.

#### 5.1.2 Constructing the SARS-CoV-2 Delta virion.

The SARS-CoV-2 Delta virion (V) model was constructed following Casalino et al. (Casalino et al., 2021) using CHARMM-GUI (Lee et al., 2016), LipidWrapper (Durrant and Amaro, 2014), and Blender (Blender Online Community, 2020), using a 350 Å lipid bilayer with an equilibrium area per lipid of 63 Å^2^ and a 100 nm diameter Blender icospherical surface mesh (Turoňová et al., 2020). The resulting lipid membrane was solvated in a 1100 Å^3^ waterbox and subjected to 4 rounds of equilibration and patching (Casalino et al., 2021). 360 M dimers and 4 E pentamers were then tiled onto the surface, followed by random placement of 29 full-length S proteins (9 open, 20 closed) according to experimentally observed S protein density (Ke et al., 2020). M and E proteins were oriented with intravirion C-termini. After solvation in a 1460 Å waterbox, the complete V model tallied >305 million atoms (Table 1). V was equilibrated for 41 ns prior to placement in the respiratory aerosol (RA) model. The equilibrated membrane was 90 nm in diameter and remains in close structural agreement with the experimental studies (Ke et al., 2020).

#### 5.1.3 Building and simulating the respiratory aerosol.

**Figure 3:**
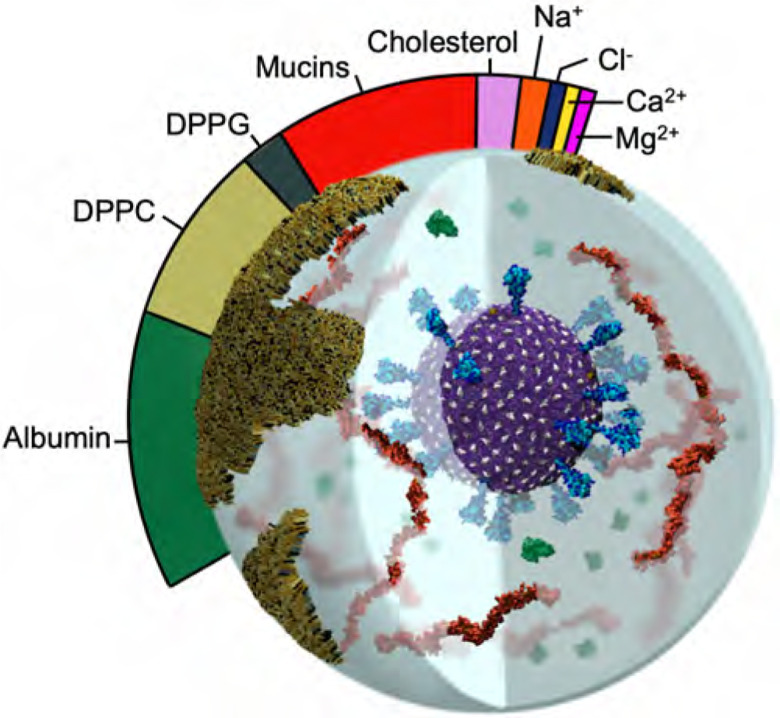
Image of RAV with relative mass ratios of RA molecular components represented in the colorbar. Water content is dependent on the relative humidity of the environment and is thus omitted from the molecular ratios.

Respiratory aerosols contain a complex mixture of chemical and biological species. We constructed a respiratory aerosol (RA) fluid based on a composition from artificial saliva and surrogate deep lung fluid recipes (Walker et al., 2021). This recipe includes 0.7 mM DPPG, 6.5 mM DPPC, 0.3 mM cholesterol, 1.4 mM Ca^2+^, 0.8 mM Mg^2+^, and 142 mM Na^+^ (Vejerano and Marr, 2018, Walker et al., 2021), human serum albumin (ALB) protein, and a composition of mucins (Fig. 3). Mucins are long polymer-like structures that are decorated by dense, heterogeneous, and complex regions of O-glycans. This work represents the first of its kind as, due to their complexity, the O-glycosylated regions of mucins have never before been constructed for molecular simulations. Two short (m_1_, m_2_, ~5 nm) and three long (m_3_, m_4_, m_5_ ~55 nm) mucin models were constructed following known experimental compositions of protein and glycosylation sequences (Hughes et al., 2019, Mariethoz et al., 2018, Markovetz et al., 2019, Symmes et al., 2018, Thomsson et al., 2005) with ROSETTA (Raveh et al., 2010) and CHARMM GUI Glycan Modeller (Jo et al., 2011). Mucin models (short and long) were solvated, neutralized by charge matching with Ca^2+^ ions, minimized, and equilibrated for 15–25 ns each (Table 1). Human serum albumin (ALB), which is also found in respiratory aerosols, was constructed from PDB 1AO6 (Sugio et al., 1999). ALB was solvated, neutralized, minimized, and equilibrated for 7ns. Equilibrated structures of ALB and the three long mucins were used in construction of the RAV with m3+m4+m5 added at 6 g/mol and ALB at 4.4 g/mol.

#### 5.1.4 Constructing the respiratory aerosolized virion model.

**Figure 4:**
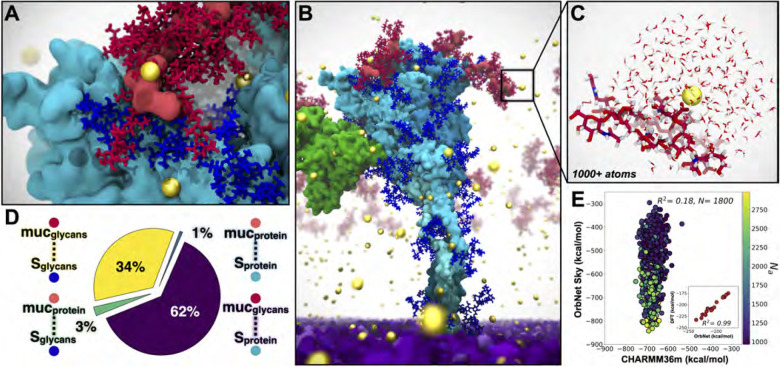
SMA system captured with multiscale modeling from classical MD to AI-enabled quantum mechanics. For all panels: S protein shown in cyan, S glycans in blue, m_1_/m_2_ shown in red, ALB in orange, Ca^2+^ in yellow spheres, viral membrane in purple. A) Interactions between mucins and S facilitated by glycans and Ca^2+^. B) Snapshot from SMA simulations. C) Example Ca^2+^ binding site from SMA simulations (1800 sites, each 1000+ atoms) used for AI-enabled quantum mechanical estimates from OrbNet Sky. D) Quantification of contacts between S and mucin from SMA simulations. E) OrbNet Sky energies vs CHARMM36m energies for each sub-selected system, colored by total number of atoms. Performance of OrbNet Sky vs. DFT in subplot (*ω*B97x-D3/def-TZVP, R^2^=0.99, for 17 systems of peptides chelating Ca^2+^ (Hu et al., 2021)). Visualized with VMD.

A 100 nm cubic box with the RA fluid recipe specified above was built with PACKMOL (Martínez et al., 2009), minimized, equilibrated briefly on TACC Frontera, then replicated to form a 300 nm cube. The RA box was then carved into a 270 nm diameter sphere. To make space for the placement of V within the RA, a spherical selection with volume corresponding to that of the V membrane + S crown (radius 734 Å) was deleted from the center of the RA. The final equilibrated V model, including surrounding equilibrated waters and ions (733 Å radius), was translated into the RA. Atom clashes were resolved using a 1.2 Å cutoff. Hydrogen mass repartitioning (Hopkins et al., 2015) was applied to the structure to improve performance. The simulation box was increased to 2800 Å per side to provide a 100 Å vacuum atmospheric buffer. The RAV simulation was conducted in an NVT ensemble with a 4 fs timestep. After minimizing, the RAV was heated to 298 K with 0.1 kcal/mol Å2 restraints on the viral lipid headgroups, then equilibrated for 1.5 ns. Finally, a cross-section of the RAV model – including and open S, m1/m2, and ALB (called the SMA system) – was constructed with PACKMOL to closely observe atomic scale interactions within the RAV model (Fig. 4).

### 5.2 Parameter evaluation with OrbNet

Comparison to quantum methods reveals significant polarization effects, and shows that there is opportunity to improve the accuracy of fixed charge force fields. For the large system sizes associated with solvated Ca^2+^-protein interaction motifs (over 1000 atoms, even in aggressively truncated systems) conventional quantum mechanics methods like density functional theory (DFT) are impractical for analyzing a statistically significant ensemble of distinct configurations (see discussion in Performance Results). In contrast, OrbNet allows for DFT accuracy with over 1000-fold speed-up, providing a useful method for benchmarking and refining the force-field simulation parameters with quantum accuracy (Christensen et al., 2021). To confirm the accuracy of OrbNet versus DFT (ωB97X-D/def2-TZVP), the inset of Fig. 4E correlates the two methods for the Ca^2+^-binding energy in a benchmark dataset of small Ca^2+^-peptide complexes (Hu et al., 2021). The excellent correlation of OrbNet and DFT for the present use case is clear from the inset figure; six datapoints were removed from this plot on the basis of a diagnostic applied to the semi-empirical GFN-xTB solution used for feature generation of OrbNet (Christensen et al., 2021).

Fig. 4E presents a comparison of the validated OrbNet method with the CHARMM36m force field for 1800 snapshots taken from the SMA MD simulations. At each snapshot, a subsystem containing a solvated Ca^2+^-protein complex was extracted (Fig. 4E), with protein bonds capped by hydrogens. For both OrbNet and the force field, the Ca^2+^-binding energy was computed and shown in the correlation plot. Lack of correlation between OrbNet and the force field identifies important polarization effects, absent in a fixed charge description. Similarly, the steep slope of the best-fit line in Fig. 4E reflects the fact that some of the configurations sampled using MD with the CHARMM36m force field are relatively high in energy according to the more accurate OrbNet potential. This approach allows us to test and quantify limitations of empirical force fields, such as lack of electronic polarization.

The practicality of OrbNet for these simulation snapshots with 1000+ atoms offers a straightforward multiscale strategy for refining the accuracy of the CHARMM36m force field. By optimizing the partial charges and other force field parameters, improved correlation with OrbNet for the subtle Ca^2+^-protein interactions could be achieved, leading to near-quantum accuracy simulations with improved configurational sampling. The calculations presented here present a proof-of-concept of this iterative strategy.

### 5.3 AI-WE simulations of Delta spike opening

**Figure 5:**
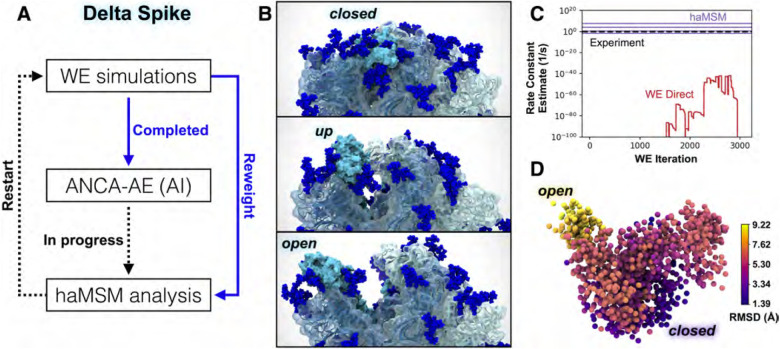
Delta-variant spike opening from WE simulations, and AI/haMSM analysis. A) The integrated workflow. B) Snapshots of the ‘down’, ‘up’, and ‘open’ states for Delta S-opening from a representative pathway generated by WE simulation, which represents ~10^5^ speedup compared to conventional MD. C) Rate-constant estimation with haMSM analysis of WE data (purple lines) significantly improves direct WE computation (red), by comparison to experimental measurement (black dashed). Varying haMSM estimates result from different featurizations which will be individually cross-validated. D) The first three dimensions of the ANCA-AE embeddings depict a clear separation between the closed (darker purple) and open (yellow) conformations of the Delta spike. A sub-sampled landscape is shown here where each sphere represents a conformation from the WE simulations and colored with the root-mean squared deviations (Å) with respect to the closed state. Visualized with VMD.

**Figure 6:**
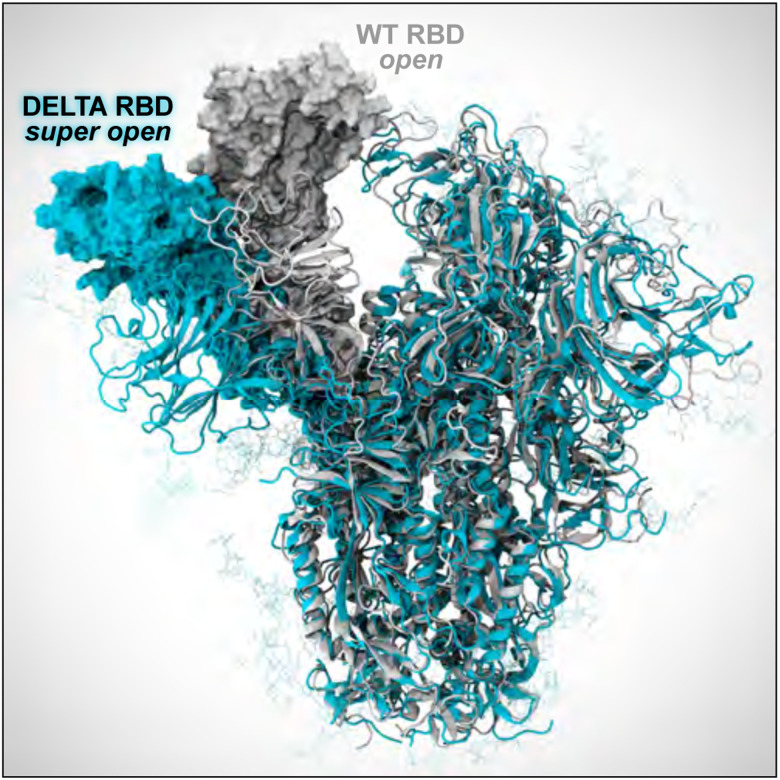
WE simulations reveal a dramatic opening of the Delta S (cyan), compared to WT S (white). While further investigation is needed, this super open state seen in the Delta S may indicate increased capacity for binding to human host-cell receptors.

While our previous WE simulations of the WT SARS-CoV-2 S-opening (Sztain et al., 2021) were notable in generating pathways for a seconds-timescale process of a massive system, we have made two critical technological advancements in the WESTPA software that greatly enhance the efficiency and analysis of WE simulations. These advances enabled striking observations of Delta-variant S opening (Figs. 5 and 6). First, in contrast to prior manual bins for controlling trajectory replication, we have developed automated and adaptive binning that enables more efficient surmounting of large barriers via early identification of “bottleneck” regions (Torrillo et al., 2021). Second, we have parallelized, memory-optimized, and implemented data streaming for the history-augmented Markov state model (haMSM) analysis scheme (Copperman and Zuckerman, 2020) to enable application to the TB-scale S-opening datasets. The haMSM approach estimates rate constants from simulations that have not yet reached a steady state (Suarez et al., 2014).

Our WE simulations generated >800 atomically detailed, Delta-variant S-opening pathways (Figs. 5B and 6) of the receptor binding domain (RBD) switching from a glycan-shielded ‘down’ to an exposed ‘up’ state using 72 *μ*s of total simulation time within 14 days using 192 NVIDIA V100 GPUs at a time on TACC’s Longhorn supercomputer. Among these pathways, 83 reach an ‘open’ state that aligns with the structure of the human ACE2-bound WT S protein (Benton et al., 2020) and 18 reach a dramatically open state (Fig. 6). Our haMSM analysis of WT WE simulations successfully provided long-timescale (steady-state) rate constants for S-opening based on highly transient information (Fig. 5C).

We also leveraged a simple, yet powerful unsupervised deep learning method called Anharmonic Conformational Analysis enabled Autoencoders (ANCA-AE) (Clyde et al., 2021) to extract conformational states from our long-timescale WE simulations of Delta spike opening (Fig. 5A,D). ANCA-AE first minimizes the fourth order correlations in atomistic fluctuations from MD simulation datasets and projects the data onto a low dimensional space where one can visualize the anharmonic conformational fluctuations. These projections are then input to an autoencoder that further minimizes non-linear correlations in the atomistic fluctuations to learn an embedding where conformations are automatically clustered based on their structural and energetic similarity. A visualization of the first three dimensions from the latent space articulates the RBD opening motion from its closed state (Fig. 5D). It is notable that while other deep learning techniques need special purpose hardware (such as GPUs), the ANCA-AE approach can be run with relatively modest CPU resources and can therefore scale to much larger systems (e.g., the virion within aerosol) when optimized.

### 5.4 D-NEMD explores pH effects on Delta spike

**Figure 7:**
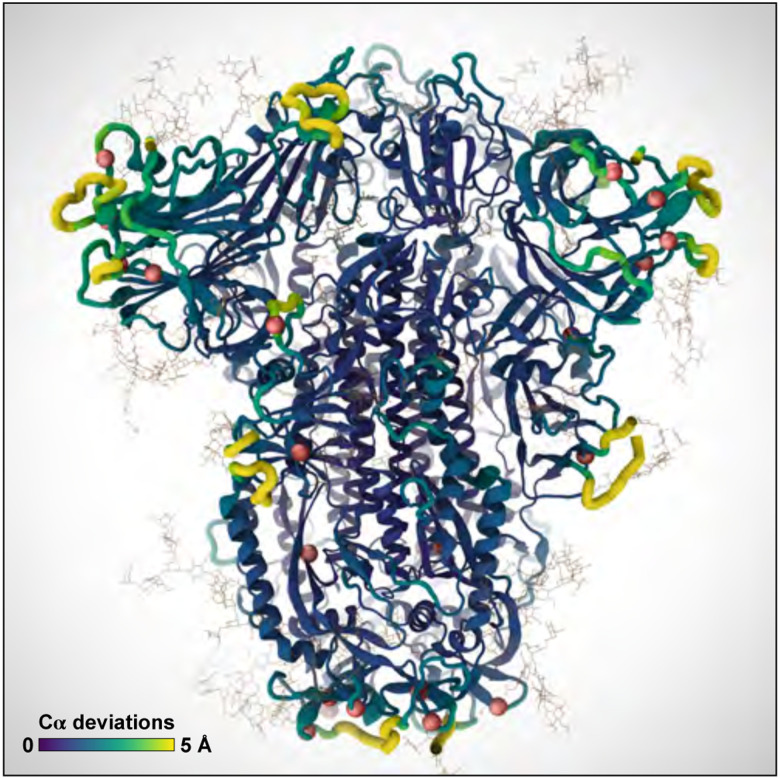
D-NEMD simulations reveal changes in key functional regions of the S protein, including the receptor binding domain, as the result of a pH decrease. Color scale and ribbon thickness indicate the degree of deviation of C*α* atoms from their equilibrium position. Red spheres indicate the location of positively charged histidines.

We performed D-NEMD simulations of the SH system with GROMACS (Abraham et al., 2015) using a ΔpH=2.0 (from 7.0 to 5.0) as the external perturbation. We ran 3 200-ns equilibrium MD simulations of SH to generate 87 configurations (29 configurations per replicate) that were used as the starting points for multiple short (10 ns) D-NEMD trajectories under the effect of the external perturbation (ΔpH=2.0). The effect of a ΔpH was modelled by changing the protonation state of histidines 66, 69, 146, 245, 625, 655, 1064, 1083, 1088, and 1101 (we note that other residues may also become protonated (Lobo and Warwicker, 2021); the D-NEMD approach can also be applied to examine those). The structural response of the S to the pH decrease was investigated by measuring the difference in the position for each C*α* atom between the equilibrium and corresponding D-NEMD simulation at equivalent points in time (Oliveira et al., 2021a), namely after 0, 0.1, 1, 5 and 10 ns of simulation. The D-NEMD simulations reveal that pH changes, of the type expected in aerosols, affect the dynamics of functionally important regions of the spike, with potential implications for viral behavior (Fig. 7). As this approach involves multiple short independent non-equilibrium trajectories, it is well suited for cloud computing. All D-NEMD simulations were performed using Oracle Cloud.

## 6 HOW PERFORMANCE WAS MEASURED

### 6.1 WESTPA

For the WE simulations of spike opening using WESTPA, we defined the time to solution as the total simulation time required to generate the first spike opening event. Spike opening is essentially impossible to observe via conventional MD. WESTPA simulations were run using the AMBER20 dynamics engine and 192 NVIDIA V100 GPUs at a time on TACC’s Longhorn supercomputer.

### 6.2 NAMD

NAMD performance metrics were collected using hardware performance counters for FLOPs/step measurements, and application-internal timers for overall simulation rates achieved by production runs including all I/O for simulation trajectory and checkpoint output. NAMD FLOPs/step measurements were conducted on TACC Frontera, by querying hardware performance counters with the rdmsr utility from Intel msr-tools^1^ and the “TACC stats” system programs.^2^ For each simulation, FLOP counts were measured for NAMD simulation runs of two different step counts. The results of the two simulation lengths were subtracted to eliminate NAMD startup operations, yielding an accurate estimate of the marginal FLOPs per step for a continuing simulation (Phillips et al., 2002). Using the FLOPs/step values computed for each simulation, overall FLOP rates were computed by dividing the FLOPs/step value by seconds/step performance data reported by NAMD internal application timers during production runs.

### 6.3 GROMACS

GROMACS 2020.4 benchmarking was performed on Oracle Cloud Infrastructure (OCI)^3^ compute shape BM.GPU4.8 consisting of 8×NVIDIA A100 tensor core GPUs, and 64 AMD Rome CPU cores. The simulation used for benchmarking contained 615,563 atoms and was run for 500,000 steps with 2 fs time steps. The simulations were run on increasing numbers of GPUs, from 1 to 8, using 8 CPU cores per GPU, running for both the production (Nose-Hoover) and GPU-accelerated (velocity rescaling) thermostats. Particle–mesh Ewald (PME) calculations were pinned to a single GPU, with additional GPUs for multi-GPU jobs used for particle–particle calculations. Performance data (ns/day and average single-precision TFLOPS, calculated as total number of TFLOPs divided by total job walltime) were reported by GROMACS itself. Each simulation was repeated four times and average performance figures reported.

## 7 PERFORMANCE RESULTS

### 7.1 NAMD performance

NAMD was used to perform all of the simulations listed in Table 1, except for the closed spike “SH” simulations described further below. With the exception of the aerosol and virion simulation, the other NAMD simulations used conventional protocols and have performance and parallel scaling characteristics that closely match the results reported in our previous SARS-CoV-2 research (Casalino et al., 2021). NAMD 2.14 scaling performance for the one billion-atom respiratory aerosol and virion simulation run on ORNL Summit is summarized in Tables 3 and 4. A significant performance challenge associated with the aerosol virion simulation relates to the roughly 50% reduction in particle density as compared with a more conventional simulation with a fully populated periodic cell. The reduced particle density results in large regions of empty space that nevertheless incur additional overheads associated with both force calculations and integration, and creates problems for the standard NAMD load balancing scheme that estimates the work associated with the cubic “patches” used for parallel domain decomposition. The PME electrostatics algorithm and associated 3-D FFT and transpose operations encompass the entire simulation unit cell and associated patches, requiring involvement in communication and reduction operations despite the inclusion of empty space. Enabling NAMD diagnostic output on a 512-node 1B-atom aerosol and virion simulation revealed that ranks assigned empty regions of the periodic cell had 66 times the number of fixed-size patches as ranks assigned dense regions. The initial load estimate for an empty patch was changed from a fixed 10 atoms to a runtime parameter with a default of 40 atoms, which reduced the patch ratio from 66 to 19 and doubled performance on 512 nodes.

### 7.2 WESTPA performance

Our time to solution for WE simulations of spike opening (to the “up” state) (Fig. 5) using the WESTPA software and AMBER20 was 14 *μ*s of total simulation time, which was completed in 4 days using 192 NVIDIA V100 GPUs at a time on TACC’s Longhorn supercomputer. For reference, conventional MD would require an expected ~5 orders of magnitude more computing. The WESTPA software is highly scalable, with nearly perfect scaling out to >1000 NVIDIA V100 GPUs and this scaling is expected to continue until the filesystem is saturated. Thus, WESTPA makes optimal use of large supercomputers and is limited by filesystem I/O due to the periodic restarting of trajectories after short time intervals.

### 7.3 AI-enhanced WE simulations

DeepDriveMD is a framework to coordinate the concurrent execution of ensemble simulations and drive them using AI models (Brace et al., 2021a, Lee et al., 2019). DeepDriveMD has been shown to improve the scientific performance of diverse problems: from protein folding to conformation of protein-ligand complexes. We coupled WESTPA to DeepDriveMD, which is responsible for resource dynamism and concurrent heterogeneous task execution (ML and AMBER). The coupled workflow was executed on 1024 nodes on Summit (OLCF), and, in spite of the spatio-temporal heterogeneity of tasks involved, the resource utilization was in the high 90%. Consistent with earlier studies, the coupling of WESTPA to DeepDriveMD results in a 100x improvement in the exploration of phase space.

### 7.4 GROMACS performance

**Figure 8:**
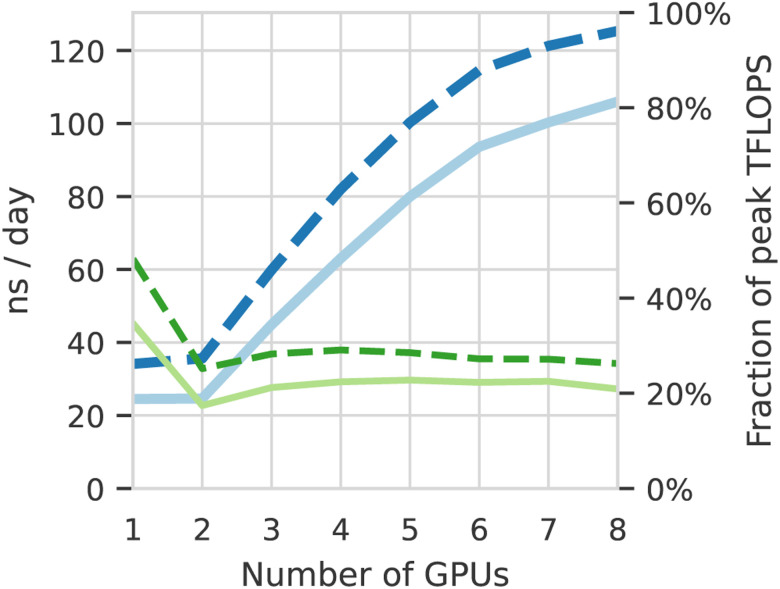
GROMACS performance across 1–8 A100 GPUs in ns/day (thicker, blue lines) and the fraction of maximum theoretical TFLOPS (thinner, green lines); production setup shown with solid line, and runs with the GPU-accelerated thermostat in dashed.

Figure 8 shows GROMACS parallelizes well across the 8 NVIDIA A100 GPUs available on each BM.GPU4.8 instance used in the *Cluster in the Cloud*^4^ running on OCI. There is a performance drop for two GPUs due to inefficient division of the PME and particle-particle tasks. Methods to address this exist for the two GPU case (Páll et al., 2020), but were not adopted as we were targeting maximum raw performance across all 8 GPUs. Production simulations achieved 27% of the peak TFLOPS available from the GPUs. Multiple simulations were run across 10 such compute nodes, enabling the ensemble to run at an average combined speed of 425 TFLOPS and sampling up to 1*μ*s/day. We note that the calculations will be able to run 20%–40% faster once the Nose-Hoover thermostat that is required for the simulation is ported to run on the GPU. Benchmarking using a velocity rescaling thermostat that has been ported to GPU shows that this would enable the simulation to extract 34% of the peak TFLOPS from the cards, enabling each node to achieve an average speed of 53.4 TFLOPS, and 125 ns/day. A cluster of 10 nodes would enable GROMACS to run at an average combined speed of over 0.5 PFLOPs, simulating over 1.2 *μ*s/day.

A significant innovation is that this power is available on demand: Cluster in the Cloud with GPU-optimized GROMACS was provisioned and benchmarked within one day of inception of the project. This was handed to the researcher, who submitted the simulations. Automatically, up to ten BM.GPU4.8 compute nodes were provisioned on-demand based on requests from the Slurm scheduler. These simulations were performed on OCI, using *Cluster in the Cloud* (Williams, 2021) to manage automatic scaling.

Cluster in the Cloud was configured to dynamically provision and terminate computing nodes based on the workload. Simulations were conducted using GROMACS 2020.4 compiled with CUDA support. Multiple simultaneous simulations were conducted, with each simulation utilizing a single BM.GPU4.8 node without multinode parallelism.

This allowed all production simulations to be completed within 2 days. The actual compute cost of the project was less than $6125 USD (on-demand OCI list price). The huge reduction in “time to science” that low-cost cloud enables changes the way that researchers can access and use HPC facilities. In our opinion, such a setup enables “exclusive on-demand” HPC capabilities for the scientific community for rapid advancement in science.

### 7.5 OrbNet performance

Prior benchmarking reveals that OrbNet provides over 1000-fold speedup compared to DFT (Christensen et al., 2021). For the calculations presented here, the cost of corresponding high quality range-separated DFT calculations (wB97X-D/def2-TZVP) can be estimated. In Fig. 4E, we consider system sizes which would require 14,000–47,000 atomic orbitals for wB97X-D/def2-TZVP, exceeding the range of typical DFT evaluations. Estimation of the DFT computational cost of the 1811 configurations studied in Fig. 4E suggests a total of 115M core-hours on NERSC Cori Haswell nodes; in contrast, the OrbNet calculations for the current study require only 100k core-hours on the same nodes. DFT cost estimates were based on extrapolation from a dataset of over 1M ChEMBL molecules ranging in size from 40 to 107 atom systems considering only the cubic cost component of DFT (Christensen et al., 2021).

## 8 IMPLICATIONS

Our major scientific achievements are:

1. We showcase an extensible AI-enabled multiscale computational framework that bridges time and length scales from electronic structure through whole aerosol particle morphology and dynamics.
2. We develop all-atom simulations of respiratory mucins, and use these to understand the structural basis of interaction with the SARS-CoV-2 spike protein. This has implications for viral binding in the deep lung, which is coated with mucins. We expect the impact of our mucin simulations to be far reaching, as malfunctions in mucin secretion and folding have been implicated in progression of severe diseases such as cancer and cystic fibrosis.
3. We present a significantly enhanced all-atom model and simulation of the SARS-CoV-2 Delta virion, which includes the hundreds of tiled M-protein dimers and the E-protein ion channels. This model can be used as a basis to understand why the Delta virus is so much more infectious than the WT or alpha variants.
4. We develop an ultra-large (1 billion+) all-atom simulation capturing massive chemical and biological complexity within a respiratory aerosol. This simulation provides the first atomic level views of virus-laden aerosols and is already serving as a basis to develop an untold number of experimentally testable hypotheses. An immediate example suggests a mechanism through which mucins and other species, e.g., lipids, which are present in the aerosol, arrange to protect the molecular structure of the virus, which otherwise would be exposed to the air-water interface. This work also opens the door for developing simulations of other aerosols, e.g., sea spray aerosols, that are involved in regulating climate.
5. We evidence how changes in pH, which are expected in the aerosol environment, may alter dynamics and allosteric communication pathways in key functional regions of the Delta spike protein.
6. We characterize atomically detailed pathways for the spike-opening process of the Delta variant using WE simulations, revealing a dramatically open state that may facilitate binding to human host cells.
7. We demonstrate how parallelized haMSM analysis of WE data can provide physical rate estimates of spike opening, improving prior estimates by many orders of magnitude. The pipeline can readily be applied to the any variant spike protein or other complex systems of interest.
8. We show how HPC and cloud resources can be used to significantly drive down time-to-solution for major scientific efforts as well as connect researchers and greatly enable complex collaborative interactions.
9. We demonstrate how AI coupled to HPC at multiple levels can result in significantly improved effective performance, e.g., with AI-driven WESTPA, and extend the reach and domain of applicability of tools ordinarily restricted to smaller, less complex systems, e.g., with OrbNet.
10. While our work provides a successful use case, it also exposes weaknesses in the HPC ecosystem in terms of support for key steps in large/complex computational science campaigns. We find lack of widespread support for high performance remote visualization and interactive graphical sessions for system preparation, debugging, and analysis with diverse science tools to be a limiting factor in such efforts.

## Figures and Tables

**Table 1:** Summary of all systems constructed in this work. See Fig 3 for illustration of aerosol construction.

^a^systems	^b^Abb.	^c^ (Å × Å × Å)	^d^N_*a*_	^e^ (ns)
^f^ M dimers	M	125 × 125 × 124	164,741	700
^f^ E pentamers	E	123 × 125 × 102	136,775	41
*Spikes:*				
^f^ (open)	S	206 × 200 × 410	1,692,444	330
^f^ (closed)	S	204 × 202 × 400	1,658,224	330
^g^ (closed, head)	SH	172 × 184 × 206	615,593	73μs
*Mucins:*				
^f^ short mucin 1	mi	123 × 104 × 72	87,076	25
^f^ short mucin 2	m2	120 × 101 × 72	82,155	25
^f^ long mucin 1	m3	810 × 104 × 115	931,778	23
^f^ long mucin 2	m4	904 × 106 × 109	997,029	15
^f^ long mucin 3	m5	860 × 111 × 113	1,040,215	18
^f^ S+m1/m2+ALB	SMA	227 × 229 × 433	2,156,689	840
^f^ Virion	V	1460 × 1460 × 1460	305,326,834	41
^f^ Resp.Aero.+Vir.	RAV	2834 × 2820 × 2828	1,016,813,441	2.42
TOTAL FLOPS			2.4 ZFLOPS	

a M, E, S, SH, and V models represent SARS-CoV-2 Delta strain.

b Abbreviations used throughout document.

c Periodic boundary dimensions.

d Total number of atoms.

e Total aggregate simulation time, including heating and equilibration runs.

f Simulated with NAMD.

g Simulated with NAMD, AMBER, and GROMACS.

**Table 2:** MD simulation floating point ops per timestep.

MD Simulation	Code	Atoms	^a^FLOPs/step
Spike, head	amber, gromacs	0.6M	62.14 gflops/step
Spike	NAMD	1.7M	43.05 gflops/step
S+m_1_/m_2_+ALB	NAMD	2.1M	54.86 gflops/step
Resp. Aero.+Vir.	NAMD	1B	25.81 tflops/step

a FLOPs/step data were computed by direct FLOP measurements from hardware performance counters for NAMD simulations, or by using the application-reported FLOP rates and ns/day simulation performance in the case of GROMACS.

**Table 3:** NAMD performance: Respiratory Aerosol + Virion, 1B atoms, 4 fs timestep w/HMR, and PME every 3 steps.

Nodes	Summit CPU + GPU	Speedup	Efficiency
256	4.18ns/day	~1.0×	~100%
512	7.68 ns/day	1.84×	92%
1024	13.64 ns/day	3.27×	81%
2048	23.10ns/day	5.53×	69%
4096	34.21 ns/day	8.19×	51%

**Table 4:** Peak NAMD FLOP rates, ORNL Summit

NAMD Simulation	Atoms	Nodes	Sim rate	Performance
Resp. Aero.+Vir.	1B	4096	34.21 ns/day	2.55 PFLOPS

## Footnotes

1
https://github.com/intel/msr-tools

2
https://github.com/TACC/tacc_stats

3
https://www.oracle.com/cloud/

4
https://cluster-in-the-cloud.readthedocs.io/

## References

[R1] Abraham M. J., Murtola T., Schulz R., Páll S., Smith J. C., Hess B., and Lindahl E.. GROMACS: High performance molecular simulations through multi-level parallelism from laptops to supercomputers. SoftwareX, 1–2:19–25, 2015. ISSN 2352–7110. doi: 10.1016/j.softx.2015.06.001. URL http://www.sciencedirect.com/science/article/pii/S2352711015000059.

[R2] Adhikari U., Mostofian B., Copperman J., Petersen A., and Zuckerman D. M.. Computational estimation of ms-sec atomistic folding times. Journal of the American Chemical Society, page 6519–6526, 2019. doi: 10.1101/427393. 30892023 PMC6660137

[R3] Bangaru S., Ozorowski G., Turner H. L., Antanasijevic A., Huang D., Wang X., Torres J. L., Diedrich J. K., Tian J.-H., Portnoff A. D., Patel N., Massare M. J., Yates J. R., Nemazee D., Paulson J. C., Glenn G., Smith G., and Ward A. B.. Structural analysis of full-length sars-cov-2 spike protein from an advanced vaccine candidate. Science, 370(6520):1089–1094, 2020. doi: 10.1126/science.abe1502. 33082295 PMC7857404

[R4] Beglov D. and Roux B.. Finite representation of an infinite bulk system: Solvent boundary potential for computer simulations. The Journal of Chemical Physics, 1994. ISSN 00219606. doi: 10.1063/1.466711.

[R5] Benton D. J., Wrobel A. G., Xu P., Roustan C., Martin S. R., Rosenthal P. B., Skehel J. J., and Gamblin S. J.. Receptor binding and priming of the spike protein of sars-cov-2 for membrane fusion. Nature, 588(7837):327–330, 2020. doi: 10.1038/s41586-020-2772-0. 32942285 PMC7116727

[R6] Bhowmik D., Gao S., Young M. T., and Ramanathan A.. Deep clustering of protein folding simulations. BMC Bioinformatics, 19(18):484, 2018. doi: 10.1186/s12859-018-2507-5. URL 10.1186/s12859-018-2507-5. 30577777 PMC6302667

[R7] Blender Online Community. Blender - a 3d modelling and rendering package, 2020. URL http://www.blender.org.

[R8] Brace A., Lee H., Ma H., Trifan A., Turilli M., Yakushin I., Munson T., Foster I., Jha S., and Ramanathan A.. Achieving 100x faster simulations of complex biological phenomena by coupling ml to hpc ensembles, 2021a.

[R9] Brace A., Salim M., Subbiah V., Ma H., Emani M., Trifa A., Clyde A. R., Adams C., Uram T., Yoo H., Hock A., Liu J., Vishwanath V., and Ramanathan A.. Stream-AI-MD: Streaming AI-Driven Adaptive Molecular Simulations for Heterogeneous Computing Platforms. Association for Computing Machinery, New York, NY, USA, 2021b. ISBN 9781450385633. URL 10.1145/3468267.3470578.

[R10] Casalino L., Gaieb Z., Goldsmith J. A., Hjorth C. K., Dommer A. C., Harbison A. M., Fogarty C. A., Barros E. P., Taylor B. C., McLellan J. S., Fadda E., and Amaro R. E.. Beyond Shielding: The Roles of Glycans in the SARS-CoV-2 Spike Protein. ACS Central Science, sep 2020. ISSN 2374–7943. doi: 10.1021/acscentsci.0c01056. URL 10.1021/acscentsci.0c01056. PMC7523240 33140034

[R11] Casalino L., Dommer A. C., Gaieb Z., Barros E. P., Sztain T., Ahn S.-H., Trifan A., Brace A., Bogetti A. T., Clyde A., Ma H., Lee H., Turilli M., Khalid S., Chong L. T., Simmerling C., Hardy D. J., Maia J. D., Phillips J. C., Kurth T., Stern A. C., Huang L., McCalpin J. D., Tatineni M., Gibbs T., Stone J. E., Jha S., Ramanathan A., and Amaro R. E.. AI-driven multiscale simulations illuminate mechanisms of SARS-CoV-2 spike dynamics. The International Journal of High Performance Computing Applications, 35(5):432–451, sep 2021. ISSN 1094–3420. doi: 10.1177/10943420211006452. URL http://journals.sagepub.com/doi/10.1177/10943420211006452. PMC8064023 38603008

[R12] Case D. A., III T. E. C., Darden T. A., Duke R. E., Giese T. J., Gohlke H., Goetz A. W., Greene D., Homeyer N., Izadi S., Kovalenko A., Lee T. S., LeGrand S., Li P., Lin C., Liu J., Luchko T., Luo D. M. R., Merz K. M., Monard G., York D. M., Kollman P. A., and Cerutti D. S.. Amber16. Publisher: University of California, San Francisco.

[R81] Chai J., Cai Y., Pang C., Wang L., McSweeney S., Shanklin J., and Liu Q.. Structural basis for SARS-CoV-2 envelope protein recognition of human cell junction protein PALS1. Nature Communications, 12(1):3433, 2021. ISSN 2041–1723. doi: 10.1038/s41467-021-23533-x. URL 10.1038/s41467-021-23533-x. PMC8187709 34103506

[R13] Christensen A. S., Sirumalla S. K., Qiao Z., O’Connor M. B., Smith D. G. A., Ding F., Bygrave P. J., Anandkumar A., Welborn M., Manby F. R., and T. F. M. I. au2. Orbnet denali: A machine learning potential for biological and organic chemistry with semi-empirical cost and dft accuracy, 2021. G. Ciccotti and M. Ferrario. Non-equilibrium by molecular dynamics: a dynamical approach. Molecular Simulation, 42(16):1385–1400, 2016. doi: 10.1080/08927022.2015.1121543. URL 10.1080/08927022.2015.1121543.

[R14] Clyde A., Galanie S., Kneller D. W., Ma H., Babuji Y., Blaiszik B., Brace A., Brettin T., Chard K., Chard R., Coates L., Foster I., Hauner D., Kertesz V., Kumar N., Lee H., Li Z., Merzky A., Schmidt J. G., Tan L., Titov M., Trifan A., Turilli M., Van Dam H., Chennubhotla S. C., Jha S., Kovalevsky A., Ramanathan A., Head M. S., and Stevens R.. High throughput virtual screening and validation of a sars-cov-2 main protease non-covalent inhibitor. bioRxiv, 2021. doi: 10.1101/2021.03.27.437323. URL https://www.biorxiv.org/content/early/2021/04/02/2021.03.27.437323. 34793155

[R15] Coleman K. K., Tay D. J. W., Sen Tan K., Ong S. W. X., Son T. T., Koh M. H., Chin Y. Q., Nasir H., Mak T. M., Chu J. J. H., Milton D. K., Chow V. T. K., Tambyah P. A., Chen M., and Wai T. K.. Viral Load of SARS-CoV-2 in Respiratory Aerosols Emitted by COVID-19 Patients while Breathing, Talking, and Singing. Clinical Infectious Diseases, aug 2021. ISSN 1058–4838. doi: 10.1093/cid/ciab691. URL 10.1093/cid/ciab691. PMC8436389 34358292

[R16] Copperman J. and Zuckerman D. M.. Accelerated estimation of long-timescale kinetics from weighted ensemble simulation via non-markovian “microbin” analysis. Journal of Chemical Theory and Computation, 16(11):6763–6775, 2020. 32990438 10.1021/acs.jctc.0c00273 PMC8045600

[R17] D’Imprima E., Floris D., Joppe M., Sánchez R., Grininger M., and Kühlbrandt W.. Protein denaturation at the air-water interface and how to prevent it. eLife, 8:e42747, 2019. ISSN 2050–084X. doi: 10.7554/eLife.42747. URL 10.7554/eLife.42747. 30932812 PMC6443348

[R18] Durrant J. D. and Amaro R. E.. LipidWrapper: An Algorithm for Generating Large-Scale Membrane Models of Arbitrary Geometry. PLoS Computational Biology, 10(7), 2014. ISSN 15537358. doi: 10.1371/journal.pcbi.1003720. PMC4102414 25032790

[R19] Fennelly K. P.. Particle sizes of infectious aerosols: implications for infection control. The Lancet Respiratory Medicine, 8(9):914–924, 2020. ISSN 22132619. doi: 10.1016/S2213-2600(20)30323-4. URL 10.1016/S2213-2600(20)30323-4. 32717211 PMC7380927

[R20] Galdadas I., Qu S., Oliveira A. S. F., Olehnovics E., Mack A. R., Mojica M. F., Agarwal P. K., Tooke C. L., Gervasio F. L., Spencer J., Bonomo R. A., Mulholland A. J., and Haider S.. Allosteric communication in class a β-lactamases occurs via cooperative coupling of loop dynamics. eLife, 10:e66567, mar 2021. ISSN 2050–084X. doi: 10.7554/eLife.66567. URL 10.7554/eLife.66567. 33755013 PMC8060031

[R21] Guvench O., Hatcher E., Venable R. M., Pastor R. W., and MacKerell A. D.. CHARMM additive all-atom force field for glycosidic linkages between hexopyranoses. Journal of Chemical Theory and Computation, 5(9):2353–2370, sep 2009. ISSN 15499618. doi: 10.1021/ct900242e. URL https://pubs.acs.org/doi/abs/10.1021/ct900242e. 20161005 PMC2757763

[R22] Han K., Venable R. M., Bryant A.-M., Legacy C. J., Shen R., Li H., Roux B., Gericke A., and Pastor R. W.. Graph–theoretic analysis of monomethyl phosphate clustering in ionic solutions. The Journal of Physical Chemistry B, 122(4):1484–1494, 2018. doi: 10.1021/acs.jpcb.7b10730. URL 10.1021/acs.jpcb.7b10730. 29293344 PMC6322214

[R23] Hopkins C. W., Le Grand S., Walker R. C., and Roitberg A. E.. Long-Time-Step Molecular Dynamics through Hydrogen Mass Repartitioning. Journal of Chemical Theory and Computation, 11(4):1864–1874, 2015. doi: 10.1021/ct5010406. URL 10.1021/ct5010406. 26574392

[R24] Hu X., Lenz-Himmer M.-O., and Baldauf C.. Better force fields start with better data – a data set of cation dipeptide interactions, 2021. 10.1038/s41597-022-01297-3 PMC9205945 35715420

[R25] Huang J. and Mackerell A. D.. CHARMM36 all-atom additive protein force field: Validation based on comparison to NMR data. Journal of Computational Chemistry, 2013. ISSN 01928651. doi: 10.1002/jcc.23354. PMC3800559 23832629

[R26] Huang J., Rauscher S., Nawrocki G., Ran T., Feig M., de Groot B. L., Grubmüller H., and MacKerell A. D.. CHARMM36m: An Improved Force Field for Folded and Intrinsically Disordered Proteins. Nature methods, 14(1):71–73, Jan. 2017. ISSN 1548–7091. doi: 10.1038/nmeth.4067. URL https://www.ncbi.nlm.nih.gov/pmc/articles/PMC5199616/. 27819658 PMC5199616

[R27] Huber G. A. and Kim S.. Weighted-ensemble Brownian dynamics simulations for protein association reactions. Biophysical Journal, 70(1):97–110, Jan. 1996. ISSN 0006–3495. URL https://www.ncbi.nlm.nih.gov/pmc/articles/PMC1224912/. 8770190 10.1016/S0006-3495(96)79552-8 PMC1224912

[R28] Hughes G. W., Ridley C., Collins R., Roseman A., Ford R., and Thornton D. J.. The MUC5B mucin polymer is dominated by repeating structural motifs and its topology is regulated by calcium and pH. Scientific Reports, 9(1):17350, 2019. ISSN 2045–2322. doi: 10.1038/s41598-019-53768-0. URL 10.1038/s41598-019-53768-0. 31758042 PMC6874590

[R29] Humphrey W., Dalke A., and Schulten K.. VMD – Visual Molecular Dynamics. J. Mol. Graphics, 14(1):33–38, 1996. doi: 10.1016/0263-7855(96)00018-5. 8744570

[R30] Jo S., Song K. C., Desaire H., MacKerell A. D., and Im W.. Glycan reader: Automated sugar identification and simulation preparation for carbohydrates and glycoproteins. Journal of Computational Chemistry, 2011. ISSN 01928651. doi: 10.1002/jcc.21886. PMC3188666 21815173

[R31] Kannan S. R., Spratt A. N., Cohen A. R., Naqvi S. H., Chand H. S., Quinn T. P., Lorson C. L., Byrareddy S. N., and Singh K.. Evolutionary analysis of the delta and delta plus variants of the sars-cov-2 viruses. Journal of Autoimmunity, 124:102715, 2021. ISSN 0896–8411. doi: 10.1016/j.jaut.2021.102715. URL https://www.sciencedirect.com/science/article/pii/S0896841121001232. 34399188 PMC8354793

[R32] Ke Z., Oton J., Qu K., Cortese M., Zila V., McKeane L., Nakane T., Zivanov J., Neufeldt C. J., Cerikan B., Lu J. M., Peukes J., Xiong X., Kräusslich H. G., Scheres S. H., Bartenschlager R., and Briggs J. A.. Structures and distributions of SARS-CoV-2 spike proteins on intact virions. Nature, pages 1–7, aug 2020. ISSN 14764687. doi: 10.1038/s41586-020-2665-2. URL https://www.nature.com/articles/s41586-020-2665-2. PMC7116492 32805734

[R33] Klauda J. B., Venable R. M., Freites J. A., O’Connor J. W., Tobias D. J., Mondragon-Ramirez C., Vorobyov I., MacKerell A. D., and Pastor R. W.. Update of the charmm all-atom additive force field for lipids: Validation on six lipid types. The Journal of Physical Chemistry B, 114(23):7830–7843, 2010. doi: 10.1021/jp101759q. URL 10.1021/jp101759q. 20496934 PMC2922408

[R34] Lee H., Turilli M., Jha S., Bhowmik D., Ma H., and Ramanathan A.. Deepdrivemd: Deep-learning driven adaptive molecular simulations for protein folding. In 2019 IEEE/ACM Third Workshop on Deep Learning on Supercomputers (DLS), pages 12–19, 2019.

[R35] Lee J., Cheng X., Swails J. M., Yeom M. S., Eastman P. K., Lemkul J. A., Wei S., Buckner J., Jeong J. C., Qi Y., Jo S., Pande V. S., Case D. A., Brooks C. L., MacKerell A. D., Klauda J. B., and Im W.. Charmm-gui input generator for namd, gromacs, amber, openmm, and charmm/openmm simulations using the charmm36 additive force field. Journal of Chemical Theory and Computation, 12(1):405–413, 2016. doi: 10.1021/acs.jctc.5b00935. URL 10.1021/acs.jctc.5b00935. 26631602 PMC4712441

[R36] Lobo V. R. and Warwicker J.. Predicted ph-dependent stability of sars-cov-2 spike protein trimer from interfacial acidic groups. Computational and Structural Biotechnology Journal, 19:5140–5148, 2021. ISSN 2001–0370. doi: 10.1016/j.csbj.2021.08.049. URL https://www.sciencedirect.com/science/article/pii/S2001037021003809. 34490059 PMC8410215

[R37] Mandala V. S., McKay M. J., Shcherbakov A. A., Dregni A. J., Kolocouris A., and Hong M.. Structure and drug binding of the SARS-CoV-2 envelope protein transmembrane domain in lipid bilayers. Nature Structural and Molecular Biology, 27(12):1202–1208, 2020. ISSN 1545–9985. URL 10.1038/s41594-020-00536-8. PMC7718435 33177698

[R38] Mariethoz J., Alocci D., Gastaldello A., Horlacher O., Gasteiger E., Rojas-Macias M., Karlsson N. G., Packer N. H., and Lisacek F.. Glycomics@ExPASy: Bridging the Gap*. Molecular and Cellular Proteomics, 17(11):2164–2176, nov 2018. ISSN 1535–9476. doi: 10.1074/mcp.RA118.000799. URL 10.1074/mcp.RA118.000799. 30097532 PMC6210229

[R39] Markovetz M. R., Subramani D. B., Kissner W. J., Morrison C. B., Garbarine I. C., Ghio A., Ramsey K. A., Arora H., Kumar P., Nix D. B., Kumagai T., Krunkosky T. M., Krause D. C., Radicioni G., Alexis N. E., Kesimer M., Tiemeyer M., Boucher R. C., Ehre C., and Hill D. B.. Endotracheal tube mucus as a source of airway mucus for rheological study. American Journal of Physiology-Lung Cellular and Molecular Physiology, 317(4):L498–L509, 2019. doi: 10.1152/ajplung.00238.2019. URL 10.1152/ajplung.00238.2019. 31389736 PMC6842913

[R40] Martínez L., Andrade R., Birgin E. G., and Martínez J. M.. Packmol: A package for building initial configurations for molecular dynamics simulations. Journal of Computational Chemistry, 30(13):2157–2164, 2009. doi: 10.1002/jcc.21224. URL https://onlinelibrary.wiley.com/doi/abs/10.1002/jcc.21224. 19229944

[R41] McCallum M., Walls A. C., Sprouse K. R., Bowen J. E., Rosen L., Dang H. V., deMarco A., Franko N., Tilles S. W., Logue J., Miranda M. C., Ahlrichs M., Carter L., Snell G., Pizzuto M. S., Chu H. Y., Van Voorhis W. C., Corti D., and Veesler D.. Molecular basis of immune evasion by the delta and kappa sars-cov-2 variants. bioRxiv, 2021. doi: 10.1101/2021.08.11.455956. URL https://www.biorxiv.org/content/early/2021/08/12/2021.08.11.455956. PMC12240541 34751595

[R42] Miller S. L., Nazaroff W. W., Jimenez J. L., Boerstra A., Buonanno G., Dancer S. J., Kurnitski J., Marr L. C., Morawska L., and Noakes C.. Transmission of SARS-CoV-2 by inhalation of respiratory aerosol in the Skagit Valley Chorale superspreading event. Indoor Air, 31(2):314–323, 2021. ISSN 16000668. doi: 10.1111/ina.12751. 32979298 PMC7537089

[R43] Noé F.. Machine Learning for Molecular Dynamics on Long Timescales, pages 331–372. Springer International Publishing, Cham, 2020. ISBN 978-3-030-40245-7. doi: 10.1007/978-3-030-40245-7_16. URL 10.1007/978-3-030-40245-7_16.

[R44] Oliveira A. S. F., Edsall C. J., Woods C. J., Bates P., Nunez G. V., Wonnacott S., Bermudez I., Ciccotti G., Gallagher T., Sessions R. B., and Mulholland A. J.. A general mechanism for signal propagation in the nicotinic acetylcholine receptor family. Journal of the American Chemical Society, 141(51):19953–19958, 2019. doi: 10.1021/jacs.9b09055. URL 10.1021/jacs.9b09055. 31805762

[R45] Oliveira A. S. F., Ciccotti G., Haider S., and Mulholland A. J.. Dynamical nonequilibrium molecular dynamics reveals the structural basis for allostery and signal propagation in biomolecular systems. The European Physical Journal B, 94(7):144, 2021a. ISSN 1434–6036. doi: 10.1140/epjb/s10051-021-00157-0. URL 10.1140/epjb/s10051-021-00157-0. 34720710 PMC8549953

[R46] Oliveira A. S. F., Shoemark D. K., Ibarra A. A., Davidson A. D., Berger I., Schaffitzel C., and Mulholland A. J.. The fatty acid site is coupled to functional motifs in the sars-cov-2 spike protein and modulates spike allosteric behaviour. bioRxiv, 2021b. doi: 10.1101/2021.06.07.447341. URL https://www.biorxiv.org/content/early/2021/06/09/2021.06.07.447341. PMC8670790 34934478

[R47] Park S. J., Lee J., Qi Y., Kern N. R., Lee H. S., Jo S., Joung I., Joo K., Lee J., and Im W.. CHARMM-GUI Glycan Modeler for modeling and simulation of carbohydrates and glycoconjugates. Glycobiology, 2019. ISSN 14602423. doi: 10.1093/glycob/cwz003. PMC6422236 30689864

[R48] Petters M. D. and Kreidenweis S. M.. A single parameter representation of hygroscopic growth and cloud condensation nucleus activity. Atmospheric Chemistry and Physics, 7(8):1961–1971, 2007. doi: 10.5194/acp-7-1961-2007. URL https://acp.copernicus.org/articles/7/1961/2007/.

[R49] Phillips J., Zheng G., Kumar S., and Kale L.. NAMD: Biomolecular simulation on thousands of processors. In Proceedings of the IEEE/ACM SC2002 Conference, Technical Paper 277, pages 1–18. IEEE Press, Baltimore, Maryland, 2002.

[R50] Phillips J. C., Braun R., Wang W., Gumbart J., Tajkhorshid E., Villa E., Chipot C., Skeel R. D., Kalé L., and Schulten K.. Scalable molecular dynamics with NAMD, 2005. ISSN 1096987X. 10.1002/jcc.20289 PMC2486339 16222654

[R51] Phillips J. C., Hardy D. J., Maia J. D. C., Stone J. E., Ribeiro J. V., Bernardi R. C., Buch R., Fiorin G., Hénin J., Jiang W., McGreevy R., Melo M. C. R., Radak B., Skeel R. D., Singharoy A., Wang Y., Roux B., Aksimentiev A., Luthey-Schulten Z., Kalé L. V., Schulten K., Chipot C., and Tajkhorshid E.. Scalable molecular dynamics on CPU and GPU architectures with NAMD. J. Chem. Phys., 153:044130, 2020. doi: 10.1063/5.0014475. 32752662 PMC7395834

[R52] Páll S., Zhmurov A., Bauer P., Abraham M., Lundborg M., Gray A., Hess B., and Lindahl E.. Heterogeneous parallelization and acceleration of molecular dynamics simulations in GROMACS. The Journal of Chemical Physics, 153(13):134110, 2020. doi: 10.1063/5.0018516. 33032406

[R53] Qiao Z., Welborn M., Anandkumar A., Manby F. R., and Miller T. F.. Orbnet: Deep learning for quantum chemistry using symmetry-adapted atomic-orbital features. The Journal of Chemical Physics, 153(12):124111, 2020. doi: 10.1063/5.0021955. URL 10.1063/5.0021955. 33003742

[R54] Raveh B., London N., and Schueler-Furman O.. Sub-angstrom modeling of complexes between flexible peptides and globular proteins. Proteins: Structure, Function, and Bioinformatics, 78(9):2029–2040, 2010. doi: 10.1002/prot.22716. URL https://onlinelibrary.wiley.com/doi/abs/10.1002/prot.22716. 20455260

[R55] Saglam A. S. and Chong L. T.. Protein–protein binding pathways and calculations of rate constants using fully-continuous, explicit-solvent simulations. Chemical Science, 10(8):2360–2372, 2019. doi: 10.1039/c8sc04811h. 30881664 PMC6385678

[R56] Salomon-Ferrer R., Götz A. W., Poole D., Le Grand S., and Walker R. C.. Routine Microsecond Molecular Dynamics Simulations with AMBER on GPUs. 2. Explicit Solvent Particle Mesh Ewald. Journal of Chemical Theory and Computation, 9(9): 3878–3888, Sept. 2013. ISSN 1549–9618. doi: 10.1021/ct400314y. 26592383

[R57] Sener M., Levy S., Stone J. E., Christensen A., Isralewitz B., Patterson R., Borkiewicz K., Carpenter J., Hunter C. N., Luthey-Schulten Z., and Cox D.. Multiscale modeling and cinematic visualization of photosynthetic energy conversion processes from electronic to cell scales. Parallel Computing, page 102698, 2021. 10.1016/j.parco.2020.102698 PMC8612599 34824485

[R58] Stone J. E., Isralewitz B., and Schulten K.. Early experiences scaling VMD molecular visualization and analysis jobs on Blue Waters. In Extreme Scaling Workshop (XSW), 2013, pages 43–50, Aug. 2013a. doi: 10.1109/XSW.2013.10.

[R59] Stone J. E., Vandivort K. L., and Schulten K.. GPU-accelerated molecular visualization on petascale supercomputing platforms. In Proceedings of the 8th International Workshop on Ultrascale Visualization, UltraVis ‘13, pages 6:1–6:8, New York, NY, USA, 2013b. ACM.

[R60] Stone J. E., Hynninen A.-P., Phillips J. C., and Schulten K.. Early experiences porting the NAMD and VMD molecular simulation and analysis software to GPU-accelerated OpenPOWER platforms. International Workshop on OpenPOWER for HPC (IWOPH’16), pages 188–206, 2016a. 10.1007/978-3-319-46079-6_14 PMC5710845 29202130

[R61] Stone J. E., Sener M., Vandivort K. L., Barragan A., Singharoy A., Teo I., Ribeiro J. V., Isralewitz B., Liu B., Goh B. C., Phillips J. C., MacGregor-Chatwin C., Johnson M. P., Kourkoutis L. F., Hunter C. N., and Schulten K.. Atomic detail visualization of photosynthetic membranes with GPU-accelerated ray tracing. Parallel Computing, 55:17–27, 2016b. doi: 10.1016/j.parco.2015.10.015. 27274603 PMC4890717

[R62] Suarez E., Lettieri S., Zwier M. C., Stringer C. A., Subramanian S. R., Chong L. T., and Zuckerman D. M.. Simultaneous computation of dynamical and equilibrium information using a weighted ensemble of trajectories. Journal of chemical theory and computation, 10(7):2658–2667, 2014. 25246856 10.1021/ct401065r PMC4168800

[R63] Sugio S., Kashima A., Mochizuki S., Noda M., and Kobayashi K.. Crystal structure of human serum albumin at 2.5 Å resolution. Protein Engineering, Design and Selection, 12(6):439–446, 06 1999. ISSN 1741–0126. doi: 10.1093/protein/12.6.439. URL 10.1093/protein/12.6.439. 10388840

[R64] Surya W., Li Y., and Torres J.. Structural model of the sars coronavirus e channel in lmpg micelles. Biochimica et Biophysica Acta (BBA) - Biomembranes, 1860(6): 1309–1317, 2018. ISSN 0005–2736. doi: 10.1016/j.bbamem.2018.02.017. URL https://www.sciencedirect.com/science/article/pii/S0005273618300580. 29474890 PMC7094280

[R65] Symmes B. A., Stefanski A. L., Magin C. M., and Evans C. M.. Role of mucins in lung homeostasis: regulated expression and biosynthesis in health and disease. Biochemical Society Transactions, 46(3):707–719, 05 2018. ISSN 0300–5127. doi: 10.1042/BST20170455. URL 10.1042/BST20170455. 29802217 PMC8359647

[R66] Sztain T., Ahn S.-H., Bogetti A. T., Casalino L., Goldsmith J. A., Seitz E., McCool R. S., Kearns F. L., Acosta-Reyes F., Maji S., Mashayekhi G., McCammon J. A., Ourmazd A., Frank J., McLellan J. S., Chong L. T., and Amaro R. E.. A glycan gate controls opening of the SARS-CoV-2 spike protein. Nature Chemistry 2021 13:10, 13(10): 963–968, aug 2021. ISSN 1755–4349. doi: 10.1038/s41557-021-00758-3. URL https://www.nature.com/articles/s41557-021-00758-3. PMC8488004 34413500

[R67] Thomsson K. A., Schulz B. L., Packer N. H., and Karlsson N. G.. MUC5B glycosylation in human saliva reflects blood group and secretor status. Glycobiology, 15(8):791–804, 04 2005. ISSN 0959–6658. doi: 10.1093/glycob/cwi059. URL 10.1093/glycob/cwi059. 15814823

[R68] Torrillo P. A., Bogetti A. T., and Chong L. T.. A minimal, adaptive binning scheme for weighted ensemble simulations. The Journal of Physical Chemistry A, 125(7): 1642–1649, 2021. doi: 10.1021/acs.jpca.0c10724. 33577732 PMC8091492

[R69] Turoňová B., Sikora M., Schürmann C., Hagen W. J. H., Welsch S., Blanc F. E. C., von Bülow S., Gecht M., Bagola K., Hörner C., van Zandbergen G., Landry J., de Azevedo N. T. D., Mosalaganti S., Schwarz A., Covino R., Mühlebach M. D., Hummer G., Krijnse Locker J., and Beck M.. In situ structural analysis of SARS-CoV-2 spike reveals flexibility mediated by three hinges. Science, page eabd5223, aug 2020. ISSN 0036–8075. doi: 10.1126/science.abd5223. URL http://science.sciencemag.org/. PMC7665311 32817270

[R70] Vejerano E. P. and Marr L. C.. Physico-chemical characteristics of evaporating respiratory fluid droplets. Journal of the Royal Society Interface, 15(139):1–10, 2018. ISSN 17425662. doi: 10.1098/rsif.2017.0939. PMC5832737 29491178

[R71] Venable R. M., Luo Y., Gawrisch K., Roux B., and Pastor R. W.. Simulations of anionic lipid membranes: Development of interaction-specific ion parameters and validation using nmr data. The Journal of Physical Chemistry B, 117(35):10183–10192, 2013. doi: 10.1021/jp401512z. URL 10.1021/jp401512z. 23924441 PMC3813009

[R72] Walker J. S., Archer J., Gregson F. K., Michel S. E., Bzdek B. R., and Reid J. P.. Accurate Representations of the Microphysical Processes Occurring during the Transport of Exhaled Aerosols and Droplets. ACS Central Science, 2021. ISSN 23747951. doi: 10.1021/acscentsci.0c01522. PMC7845015 33532579

[R73] Walls A. C., Park Y. J., Tortorici M. A., Wall A., McGuire A. T., and Veesler D.. Structure, Function, and Antigenicity of the SARS-CoV-2 Spike Glycoprotein. Cell, 181(2): 281–292.e6, apr 2020. ISSN 10974172. doi: 10.1016/j.cell.2020.02.058. 32155444 PMC7102599

[R74] Wang C. C., Prather K. A., Sznitman J., Jimenez J. L., Lakdawala S. S., Tufekci Z., and Marr L. C.. Airborne transmission of respiratory viruses. Science, 373(6558):eabd9149, 2021. ISSN 0036–8075. doi: 10.1126/science.abd9149. 34446582 PMC8721651

[R75] Warwicker J.. A model for pH coupling of the SARS-CoV-2 spike protein open/closed equilibrium. Briefings in Bioinformatics, 22(2):1499–1507, 02 2021. ISSN 1477–4054. doi: 10.1093/bib/bbab056. URL 10.1093/bib/bbab056. 33634309 PMC8108619

[R76] Williams M.. Cluster in the Cloud, October 2021. URL https://cluster-in-the-cloud.readthedocs.io.

[R77] Wrapp D., Wang N., Corbett K. S., Goldsmith J. A., Hsieh C.-L., Abiona O., Graham B. S., and McLellan J. S.. Cryo-EM structure of the 2019-nCoV spike in the prefusion conformation. Science (New York, N.Y.), 1263(March):1260–1263, 2020. ISSN 1095–9203. doi: 10.1126/science.abb2507. URL http://www.ncbi.nlm.nih.gov/pubmed/32075877. PMC7164637 32075877

[R78] Zimmerman M. I., Porter J. R., Ward M. D., Singh S., Vithani N., Meller A., Mallimadugula U. L., Kuhn C. E., Borowsky J. H., Wiewiora R. P., and Sars-cov-2 simulations go exascale to predict dramatic spike opening and cryptic pockets across the proteome. Nature Chemistry, 13(7):651–659, 2021. doi: 10.1038/s41557-021-00707-0. PMC8249329 34031561

[R79] Zuckerman D. M. and Chong L. T.. Weighted Ensemble Simulation: Review of Methodology, Applications, and Software. Annual Review of Biophysics, 46:43–57, 2017. ISSN 1936–1238. doi: 10.1146/annurev-biophys-070816-033834. PMC5896317 28301772

[R80] Zwier M. C., Adelman J. L., Kaus J. W., Pratt A. J., Wong K. F., Rego N. B., Suárez E., Lettieri S., Wang D. W., Grabe M., Zuckerman D. M., and Chong L. T.. WESTPA: An Interoperable, Highly Scalable Software Package for Weighted Ensemble Simulation and Analysis. Journal of Chemical Theory and Computation, 11(2):800–809, Feb. 2015. doi: 10.1021/ct5010615. URL 10.1021/ct5010615. 26392815 PMC4573570

